# Differences in the Composition of Archaeal Communities in Sediments from Contrasting Zones of Lake Taihu

**DOI:** 10.3389/fmicb.2016.01510

**Published:** 2016-09-21

**Authors:** Xianfang Fan, Peng Xing

**Affiliations:** ^1^State Key Laboratory of Lake Science and Environment, Nanjing Institute of Geography and Limnology (CAS)Nanjing, China; ^2^State Key Laboratory of Soil and Sustainable Agriculture, Institute of Soil Science (CAS)Nanjing, China

**Keywords:** Archaea, sediment, Lake Taihu, *Cyanobacteria*, macrophyte, difference

## Abstract

In shallow lakes, different primary producers might impact the physiochemical characteristics of the sediment and the associated microbial communities. Until now, little was known about the features of sediment Archaea and their variation across different primary producer-dominated ecosystems. Lake Taihu provides a suitable study area with cyanobacteria- and macrophyte-dominated zones co-occurring in one ecosystem. The composition of the sediment archaeal community was assessed using 16S rRNA gene amplicon sequencing technology, based on which the potential variation with respect to the physiochemical characteristics of the sediment was analyzed. *Euryarchaeota* (30.19% of total archaeal sequences) and *Bathyarchaeota* (28.00%) were the two most abundant phyla, followed by *Crenarchaeota* (11.37%), *Aigarchaeota* (10.24%) and *Thaumarchaeota* (5.98%). The differences found in the composition of the archaeal communities between the two zones was significant (*p* = 0.005). Sediment from macrophyte-dominated zones had high TOC and TN content and an abundance of archaeal lineages potentially involved in the degradation of complex organic compounds, such as the order *Thermoplasmatales*. In the area dominated by *Cyanobacteria*, archaeal lineages related to sulfur metabolism, for example, *Sulfolobales* and *Desulfurococcales*, were significantly enriched. Among *Bathyarchaeota*, subgroups MCG-6 and MCG-15 were significantly accumulated in the sediment of areas dominated by macrophytes whereas MCG-4 was consistently dominant in both type of sediments. The present study contributes to the knowledge of sediment archaeal communities with different primary producers and their possible biogeochemical functions in sediment habitats.

## Introduction

Lake Taihu is a large (2338 km^2^) shallow (1.9 m mean depth) eutrophic freshwater lake in China (Qin, 2008). It is an advantageous location with two alternative states of equilibrium in one system: a turbid state dominated by *Cyanobacteria* and a clear state dominated by aquatic macrophytes (Scheffer et al., 1993; Scheffer and van Nes, 2007). The cyanobacteria-dominated lake zone mainly includes the western shores and northern bays, such as Meiliang Bay and Zhushan Bay (Luo et al., 2014) where phytoplankton diversity has decreased since early 1980s, but cyanobacteria populations (*Microcystis* and *Anabaena*) have increased and can comprise 85% of summer phytoplankton biomass (Chen et al., 2003; Guo, 2007; Liu et al., 2011). The macrophyte-dominated lake zones are mainly at the eastern part of the lake, including East Taihu Bay, Xukou Bay, and Gonghu Bay (Luo et al., 2014, 2016). The aquatic macrophytes mainly include two types: floating-leaf vegetation, with *Nymphoides* *peltatum* as the dominant species, and submerged vegetation, with *Potamogeton malaianus* as the dominant species (Zhao et al., 2012).

The sediment heterogeneity induced by different primary producers may influence the respective sediment microbial communities. The sediment environment is an important site where organic matter is degraded and transformed. Both phytoplankton and macrophytes are important sources of sediment organic matter during their sedimentation and biodegradation (Lauster et al., 2006). Therefore, different dominant primary producers could indicate differences in the quality of organic matter (Torremorell et al., 2015). Compared with algae-source carbon, macrophyte-derived carbon is believed to be more refractory to microbial consumption. The less efficient metabolic processes and carbon cycling in the macrophyte-dominated area induce the accumulation of recalcitrant organic matter within sediment (Rejmánková and Houdková, 2006; Zhang et al., 2013). Moreover, the degradation of settled phytoplankton bloom biomass directly contributes to the higher availability of P, Fe, and S and the stimulated biogeochemical cycling of such elements (Handley et al., 2012; Zhu et al., 2013; Han et al., 2015). In addition, the various processes of organic matter transformation may impact the physiochemical characteristics of sediment (Hinsinger, 2001; Chen et al., 2014). Sediment heterogeneity has been shown to induce significant changes in the composition of bacterial, methanogen and ammonia-oxidizing prokaryote communities in shallow lake sediments (Wu et al., 2010; Fan and Wu, 2014; Chen et al., 2015). However, almost nothing is known about the composition of archaeal communities in sediments from lake areas dominated by either macrophytes or planktonic cyanobacteria.

Analysis of 16S rRNA gene sequences from a wide range of environmental samples has revealed that Archaea are not only extreme prokaryotes but also ubiquitous and far more abundant than previously estimated (Cavicchioli, 2011; Lloyd et al., 2013a). New genomes and 16S rRNA gene sequences have dramatically expanded in recent years, with several new phylum-level lineages and two proposed superphyla, ‘TACK’ (including *Thaumarchaeota, Aigarchaeota, Crenarchaeota, Korarchaeota, Bathyarchaeota, Lokiarchaeota*) and ‘DPANN’ (including *Diapherotrites, Parvarchaeota, Aenigmarchaeota, Nanoarchaeota, Nanohaloarchaeota, Pacear chaeota, Woesearchaeota, Micrarchaeota*) (Eme and Doolittle, 2015). Culture-independent approaches, such as metagenomics and single-cell genomics, shed light on the metabolic diversity of uncultured archaeal lineages (Offre et al., 2013). Archaea have evolved various capacities for energy metabolisms using organic and/or inorganic electron donors and acceptors. Also many archaeal species can fix carbon from inorganic sources and influence the dynamic equilibrium of greenhouse gasses. The abundant biomass, ubiquity and versatile metabolisms of Archaea indicate that they might play important roles in the biogeochemical cycles of C, N, and S in lake sediment although less information is available in comparison to bacterial counterparts.

Since little was known about the features of sediment Archaea and their variation across different primary producer-dominated ecosystems, eleven sampling sites were established around Lake Taihu, with six sites in the cyanobacteria-dominated zone and five sites in the macrophyte-dominated zone. The archaeal community composition was investigated using 16S rRNA gene amplicon sequencing methodology. In particular, we were interested in the potential differences in the composition of archaeal communities between sediments collected at these two contrasting lake zones, identifying both the dominant groups in each sediment and the main environmental drivers responsible for such differences.

## Materials and Methods

### Site Description and Sample Collection

**FIGURE 1:**
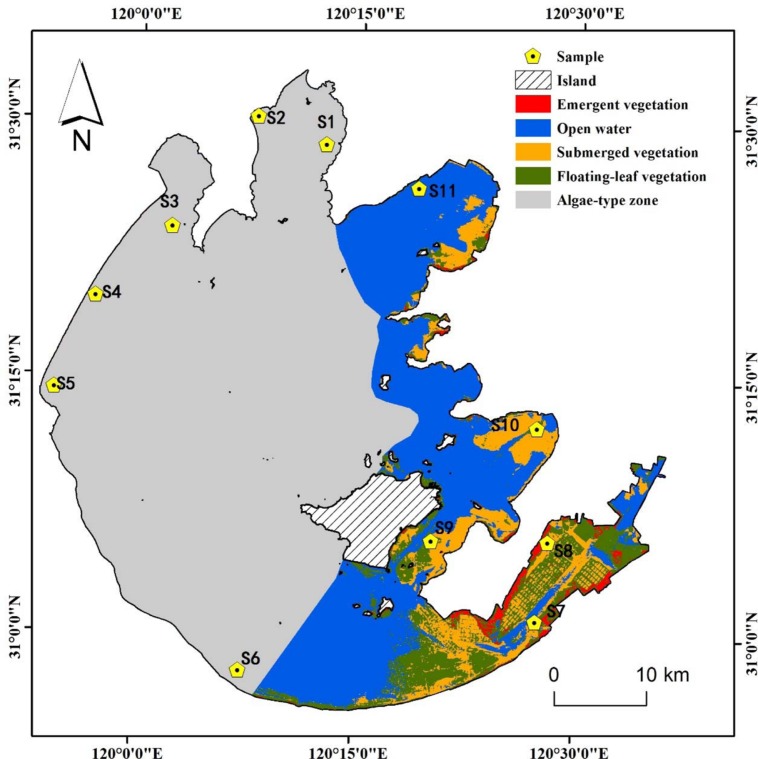
**Location of sampling sites in two featured regions of Lake Taihu.** Sites S1 to S6 denotes cyanobacteria-dominated zones and sites S7 to S11 denotes macrophyte-dominated zones.

Eleven sampling sites (S1–S11) were selected around Lake Taihu (30° 55.667′ – 31° 32.967′ N, 119° 52.533′ – 120° 36.167′ E) (**Figure 1**). Sampling sites S1–S6 were in the cyanobacteria-dominated zone, and sampling sites S7–S11 were in the aquatic macrophyte-dominated zone (Luo et al., 2014). Sediment cores (8.6 cm inner diameter, 25 cm length) were collected from the 11 sampling sites on July 9, 2010. Within 4 h, the sediment cores, stored on ice, were transported by car to the Nanjing Institute of Geography and Limnology. After arriving at the laboratory, the upper 0–20 cm of sediment was homogenized as thoroughly as possible. Then, samples for DNA extraction were stored at -20°C, and those for soil chemical property analysis were stored at 4°C.

### Physiochemical Analysis

The physicochemical properties of the overlying water were investigated using a Multi-Parameter Water Quality Sonde (YSI 6600, USA). The chlorophyll a (Chl*a*) in the sediment was determined by UV-Vis spectrophotometry (HP8452, Canada). The TC/TN ratio of the sediment was determined by an elemental analyzer (EURO EA3000, Italy). Total phosphorus (TP) was analyzed using the molybdenum antimony resistance-colorimetric method. Total nitrogen (TN) was analyzed by the Kieldahl method. Total organic carbon (TOC) was analyzed using the potassium dichromate oxidation-ferrous sulfate titrimetry method. Loss on ignition (LOI) was analyzed by heating at 550°C for 2 h. The TP, TN, TOC, and LOI of the sediment were analyzed according to Bao (2000).

### DNA Extraction

Before DNA extraction, the sediment samples were freeze-dried at -53°C by a freeze dryer (Labconco FreeZone Triad 2.5 L, USA), and 0.5 g of the dried sediment was used to extract DNA, using a FastDNA spin kit for soil (MP Biomedicals LLC, Solon, OH, USA). The quality and size of the DNA were checked by electrophoresis on 1% agarose gels, and the size of the main DNA fraction recovered ranged from 4 to 20 kb.

### Real-Time Quantitative Polymerase Chain Reaction (PCR)

The abundance of Bacteria and Archaea was estimated by quantitative PCR for fragments of 16S rRNA gene using the bacterial domain-specific primer set Eub341F and Eub515R (Simmons et al., 2007) and the archaeal domain-specific primer set Arch349F and Arch806R (Takai and Horikoshi, 2000), respectively. Quantitative PCR was performed using a CFX96 Optical Real-Time Detection System (Bio-Rad Laboratories, Inc. Hercules, CA, USA). Quantification was based on the fluorescence intensity of the SYBR Green dye, which binds to double-stranded DNA. Real-time PCR standard curves were generated as described by Jia and Conrad (2009). The target PCR products were purified and ligated into the pGEM-T vector (Promega, Madison, WI, USA) according to the manufacturer’s instructions. Plasmids were transformed into *Escherichia coli* JM 109 component cells (Takara, Dalian, China). Plasmid DNA of individual clones cultured in Luria-Bertani medium was extracted, purified and quantified. Then equal amounts of plasmid DNA from eleven representative clones for sample S1 to S11 were mixed and quantified again. A dilution series of the stock mixture of plasmid DNA inserted by bacterial 16S rRNA gene was made to generate a standard curve covering six orders of magnitude from 3.0 × 10^4^ to 3.0 × 10^11^ copies of template per assay. The standard curve of the archaeal 16S rRNA gene covered six orders of magnitude as well, from 1.2 × 10^4^ to 1.2 × 10^10^ copies of template per assay. A blank was always run with water as a template instead of extracted soil DNA. The real-time PCR assay was carried out in a 20 μl reaction volume containing 10 μl of SYBR^®^ Premix Ex Taq (TaKaRa Biotech, Dalian, China), 0.25 μM of each primer and 1.0 μl of template. The specificity of amplification was verified by melting curve analysis, which always resulted in a single peak. The melting temperature of bacterial 16S rRNA gene was 87.5°C, and the amplification efficiency was 96.6% with an *R^2^* value of 0.996. The melting temperature of archaeal 16S rRNA gene was 87°C, and the amplification efficiency was 97.4% with *R^2^* value of 0.998. Real-time PCR was performed in triplicate.

### PCR Amplification, Sequencing, and Sequence Data Processing

The extracted DNA was amplified with the archaeal domain-specific primer set 519f (5′-CAGCMGCCGCGGTAA-3′) (Øvreås et al., 1997) with barcode and 915r (5′-GTGCTCCCCCGCCAATTCCT-3′) (Stahl and Amann, 1991). The protocol and conditions used were similar to those outlined by Coolen et al. (2004) for PCR. The amplicons were purified using a Gel Extraction Kit (Takara Bio, Dalian, China). The size of the amplicons was confirmed by electrophoresis on 2% agarose gels. The purity and quantity of the amplicons were assessed using a Nanodrop ND-1000 UV-Vis Spectrophotometer (NanoDrop Technologies, Wilmington, DE, USA). The amplicons for different samples were then pooled in equimolar ratios for sequencing analysis. Library preparation and DNA sequencing on the Illumina MiSeq platform using the 2 × 250 bp paired-end protocol (Illumina, Inc., San Diego, CA, USA) were performed at Shanghai BIOZERON Biotechnology Co., Ltd. (Shanghai, China). The raw sequence data were submitted to the ENA database (PRJEB10388).

Trimmomatic software was used to process the raw sequence data for quality control (Bolger et al., 2014). The PE reads were overlapped to assemble the final tag sequences with the minimum overlap length as 10 bp. We removed all of the sequences that contained more than one ambiguous base “N,” those that contained any errors in the forward or reverse primers, and those with more than a 0.2 mismatch ratio within the overlap region. The tail base of reads with quality values below 20 was filtered, and variable tags (overlap length minus primers and barcodes) that were shorter than 50 bp were also removed. The obtained clean sequences were then analyzed using QIIME software (Caporaso et al., 2010). The clean sequences were screened for chimeras using Usearch (Edgar et al., 2011). Then, operational taxonomic unit (OTU) grouping was performed in Usearch (97% cutoff). Taxonomic assignment of OTU representative sequences was done against the SILVA database (Quast et al., 2013, release_123) at 80% similarity using the RDP Classifier (Cole et al., 2014). After the taxonomic assignment, a data set comprising only the archaeal taxa was selected from all of the high-quality sequences.

Two hundred and twenty reference sequences of the 21 known *Bathyarchaeota* (previously named Miscellaneous Crenarchaeotal Group, MCG) subgroups (Kubo et al., 2012; Fillol et al., 2016) were aligned in SILVA Incremental Aligner (SINA, Pruesse et al., 2012) and imported into ARB software environment (Ludwig et al., 2004). A 50% conserved filter considering 88 well-aligned reference sequences (>940 bp) was implemented. The *Bathyarchaeota* backbone tree was built with RAxML that estimates large phylogenies by maximum likelihood and bootstrapped for 1000 repetitions. Bathyarchaeotal sequences (∼405 bp) obtained in this study were aligned in SINA and added to the tree by parsimony criteria without allowing changes in the general tree topology. Circular tree was edited with the online tool Interactive Tree of Live (iTOL, Letunic and Bork, 2007).

### Statistical Analysis

The normality of data was tested by the Kolmogorov–Smirnov test. If the distribution of data was normal, the *t*-test was selected to evaluate the significance of differences between the cyanobacteria-dominated zone and the macrophyte-dominated zone. Otherwise, the Mann–Whitney test was selected. The above analyses were performed using SPSS version 16.0 with significant level 0.05 (SPSS Inc., Chicago, IL, USA).

To perform downstream analyses, the OTU matrix was normalized by setting the uniform sequence number to 22965 for each sample. The diversity of the archaeal community was determined by the Shannon and Faith’s phylogenetic diversity (PD) indices, and its richness was indicated by the Chao index. Moreover, the coverage of diversity and the number of observed OTUs were provided. All the above α-diversity analysis was done in QIIME pipeline (Caporaso et al., 2010).

To further quantify the observed differences, non-parametric statistics based on the Bray–Curtis dissimilarity index were performed using the OTU data. An analysis of similarities (ANOSIM) was performed to test whether there was a significant difference in the archaeal community composition between the cyanobacteria-dominated and macrophyte-dominated zones. To visually interpret the community dissimilarity at the OTU level, non-metric multidimensional scaling ordination (NMDS) was carried out. To investigate the relationship between the archaeal species (i.e., OTU) and the physicochemical factors, the multivariate constrained ordination method was used. Detrended correspondence analysis (DCA) showed that the largest axis length was 2.41. Consequently, redundancy analysis (RDA) was selected, and the significance of the total physicochemical factors was tested with Monte Carlo permutations (permu = 999). Environmental factors were selected by the function ‘vif.cca’, and environmental factors with vif >20 were removed from the subsequent analysis. TOC, LOI, Chl*a*, TP and TC/TN were selected for RDA. The above analyses were conducted in R for statistical computing (R Development Core Team, 2013) using the **vegan** package for ANOSIM, NMDS and RDA (Oksanen et al., 2013).

Linear discriminant analysis (LDA) effect size (LEfSe) was used to identify the taxa characterizing the differences between the zones (Segata et al., 2011). The LEfSe analysis was performed on a website^1^. Differential features were identified at the species level. The zone groups were used as the class of subjects (no subclass). The LEfSe analysis was performed under the following conditions: the alpha value for the factorial Kruskal–Wallis test among classes was <0.05, and the threshold on the logarithmic LDA score for discriminative features was 4.0.

## Results

### Physicochemical Properties of the Sediment Samples

The concentrations of TOC and TN in the cyanobacteria-dominated zone sediment were significantly lower than those in the macrophyte-dominated zone sediment, both with *p* values of 0.003 (**Table 1**; **Supplementary Table S1**). However, the variables Chl*a*, TP, LOI and TC/TN were not significantly different between the sediments in the two zones. The concentration of TP tended to be higher in the cyanobacteria-dominated zone sediment, whereas the concentrations of Chl*a*, LOI and TC/TN tended to be higher in the macrophyte-dominated zone sediment. For the physiochemical factors of water column, no significant difference was detected between the two typical regions (**Supplementary Figure S1**).

**Table 1:** Physicochemical characteristics of the sediment samples from two major regions in Lake Taihu (Standard deviation of replicates are in brackets; ^∗∗^ indicated significance at 0.01 level).

Zone	Sample	Chl*a* (ug/kg)	TOC^∗∗^ (g/kg)	TN^∗∗^ (g/kg)	TP (g/kg)	LOI (%)	TC/TN ratio
Cyanobacteria- dominated zone	S1	504.57	13.908	1.39	1.33	7.05	7.4
	S2	579.64	10.797	1.08	1.18	5.94	8.6
	S3	703.42	9.961	1.00	0.65	1.04	7.2
	S4	602.12	11.01	1.10	0.75	6.55	8.5
	S5	317.69	8.404	0.84	0.66	2.01	7.9
	S6	157.43	10.183	1.02	0.73	3.73	10.9
	Mean (SD)	477.48 (202.84)	10.71 (1.82)	1.07 (0.18)	0.88 (0.29)	4.39 (2.51)	8.4 (1.3)
Macrophyte- dominated zone	S7	328.14	17.86	1.78	0.47	4.94	15.2
	S8	578.57	22.18	2.22	1.26	12.97	7.82
	S9	551.95	15.07	1.51	0.61	0.75	9.52
	S10	708.54	15.19	1.52	0.56	30.47	10.0
	S11	702.57	14.20	1.42	0.88	6.14	8.5
	Mean (SD)	573.95 (154.58)	16.90 (3.25)	1.69 (0.33)	0.76 (0.32)	11.05 (11.71)	10.2 (2.9)


### Community Composition of Archaea in the Cyanobacteria-Dominated and Macrophyte-Dominated Zones

**FIGURE 2:**
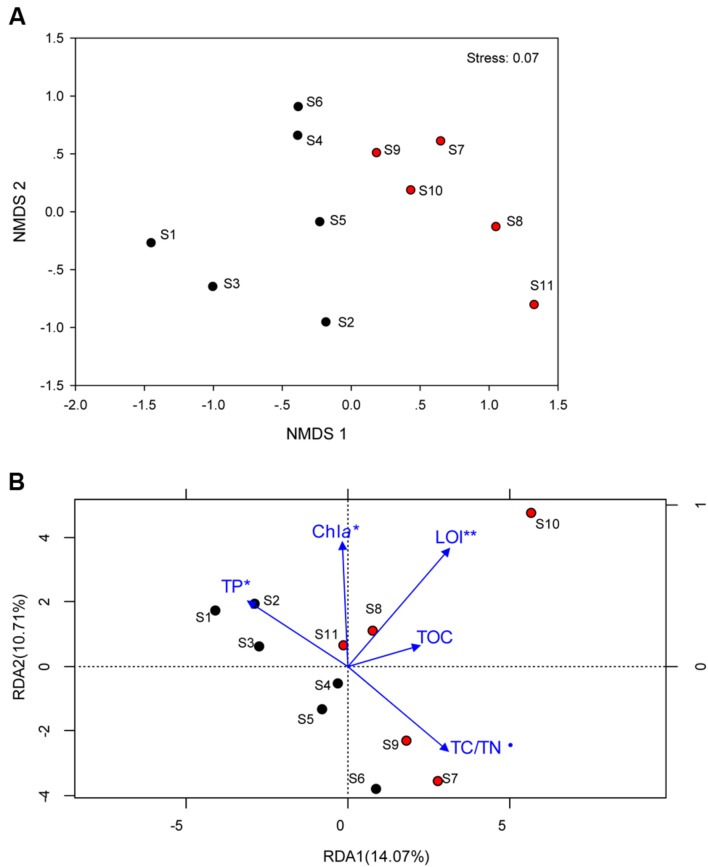
**(A)** Non-metric multidimensional scaling ordination (NMDS) plot of the archaeal communities; **(B)** Redundancy analysis (RDA) of the archaeal community composition with sediment physichemical charateristics (blue arrows) for the 11 sampling sites. Samples in the cyanobacteria-dominated zone was marked by black filled cycles; Samples in the macrophyte-dominated zone was marked by red filled cycles; those physichemical charateristics which are significant correlation with the dynamics of archaeal were marked with ⋅ for 0.1 level, ^∗^for 0.05 level and ^∗∗^ for 0.01 level.

Total *Archaea*, which approximately equivalent to 1/10 of bacterial number were not significantly different between the two regions, whereas it tended to be higher in sediment dominated by *Cyanobacteria* (**Supplementary Figure S2**). The coverage estimate indicated that the archaeal 16S rRNA gene library for each sample was sufficiently large to capture the total estimated OTUs (**Table 2**; **Supplementary Table S1**). The richness and diversity of the archaeal community tended to be higher in the cyanobacteria-dominated zone sediment, although there were no significant variations between the zones based on the Chao index or Shannon and Faith’s PD indices. The differences in archaeal community composition at the OTU level are qualitatively displayed in an NMDS plot, where relative similarity among the sampled sediments is indicated by the clustering of zone-specific data points (**Figure 2A**). A global ANOSIM comparison further indicated a statistically significant difference in overall archaeal community composition at the OTU level between the zone sediments (*p* = 0.005). The RDA results showed that the first axis (RDA1) explains 14.07% of the archaeal community variance and that the second axis (RDA2) explains 10.71% (**Figure 2B**). The results of Monte Carlo permutations indicated that the environmental factors LOI (*p* = 0.005), TP (*p* = 0.032), Chl*a* (*p* = 0.034) and TC/TN (*p* = 0.078) were significantly correlated with the archaeal community distribution. TP was significantly correlated with archaeal communities in the cyanobacteria-dominated zone, while LOI and TC/TN were significantly correlated with archaeal communities in the macrophyte-dominated zone sediment, as indicated by the direction of the environmental factor vectors.

**Table 2:** Alpha-diversity index for archaeal communities of the sediment samples (Standard deviation of replicates are in brackets; no significant difference was found for each variable).

Zone	Sample	OTUs	Chao	Shannon	PD	Coverage
Cyanobacteria-dominated zones	S1	1640	2128	4.94	114.11	0.98


	S2	1438	1709	4.65	136.18	0.98
	S3	1371	1604	4.57	127.03	0.98
	S4	1543	2055	4.54	116.87	0.98
	S5	1318	1525	4.54	103.52	0.99
	S6	1333	1772	4.42	101.04	0.98
	Mean (SD)	1441 (128)	1799 (243)	4.61 (0.18)	116.46 (13.50)	0.98 (0.38)
Macrophyte-dominated zones	S7	1380	2015	4.45	105.24	0.98


	S8	1069	1177	4.25	85.99	0.99
	S9	1573	2031	4.56	117.94	0.98
	S10	1471	1892	4.53	120.04	0.98
	S11	995	1235	3.80	92.65	0.99
	Mean (sd)	1298 (253)	1670 (427)	4.32 (0.31)	104.37 (15.05)	0.98 (0.61)


**FIGURE 3:**
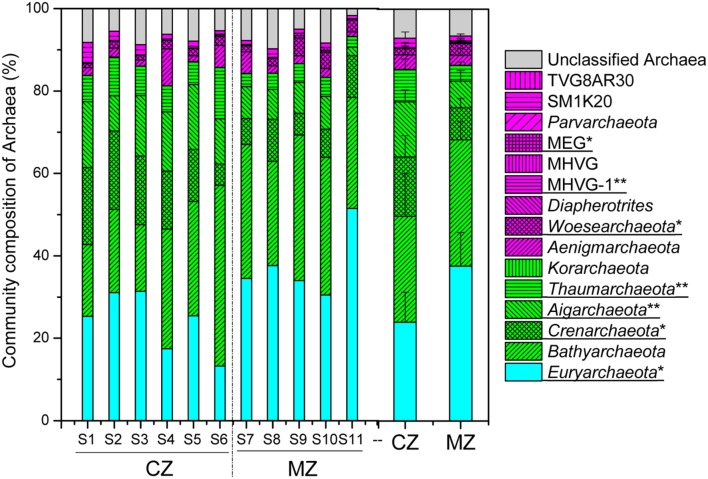
**The overview of archaeal community composition at phylum level (CZ, cyanobacteria-dominated zone; MZ, macrophyte-dominated zone; Green shadow denoting the TACK supergroup; magenta denoting the DPANN supergroup; MHVG, Marine Hydrothermal Vent Group; MHVG-1, Marine Hydrothermal Vent Group 1; MEG, Miscellaneous Euryarchaeotic Group; those phyla with significant difference between the two regions were underlined with ^∗^ for 0.05 level and ^∗∗^ for 0.01 level)**.

The results of taxonomic assignment at the phylum level indicated that seven phyla had relative abundance >1% of total archaeal sequences in all samples, including *Euryarchaeota* (30.19%), *Bathyarchaeota* (28.00%), *Crenarchaeota* (11.37%), *Aigarchaeota* (10.24%), *Thaumarchaeota* (5.98%), *Aenigmarchaeota* (3.04%) and *Woesearchaeota* (2.20%) (**Figure 3**; **Supplementary Table S1**). The relative abundance of phyla *Crenarchaeota* (*p* = 0.027), *Aigarchaeota* (*p* = 0.003) and *Thaumarchaeota* (*p* = 0.009) was significantly higher in the cyanobacteria-dominated zone sediment, whereas the relative abundance of phyla *Euryarchaeota* (*p* = 0.017) and *Woesearchaeota* (*p* = 0.048) were significantly higher in sediments collected at lake zones dominated by macrophytes (**Figure 3**).

**FIGURE 4:**
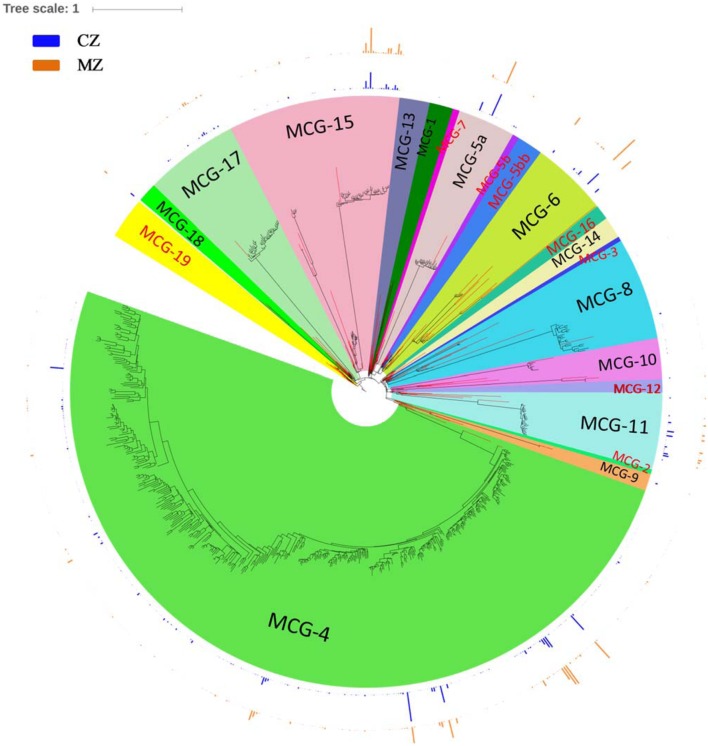
**Phylogenetic maximum-likelihood (ML) tree was built up for the total 605 representative *Bathyarchaeota* SSU rRNA gene sequences (black branches) obtained in the study with Terrestrial Hot Springs Crenarchaeotal Group as out-group.** Sequences for typical *Bathyarchaeota* subgroups according to the nomenclatures promoted by Kubo et al. (2012) and Fillol et al. (2016) are used as major references for constructing the phylogenetic tree (red branches). Outer bar charts indicate the ratio of average sequence number belong to a certain OTU divided by the total number of sequences in a given sediment type. ML tree was built in ARB and edited with online tool iTOL (http://itol.embl.de/). CZ, cyanobacteria-dominated zone; MZ, macrophyte-dominated zone; subgroup name in black fonts indicates it contains bathyarchaeotal sequences generated in this study.

In our study, sequences affiliated to *Bathyarchaeota* clustered into 605 OTUs, which account for ∼28.00% of the total sequences and 15.98% of the total OTUs. Affiliation of all these sequences to the 21 recently defined subgroups (Fillol et al., 2016) resulted in sequences clustering into 13 subgroups, namely, MCG-1, MCG-4, MCG-5a, MCG-6, MCG-8, MCG-9, MCG-10, MCG-11, MCG-13, MCG-14, MCG-15, MCG-17, and MCG-18 (**Figure 4**). The top five abundant *Bathyarchaeota* subgroups accounting for >5% of the total bathyarchaeotal sequences were MCG-4 (53.98%, 37,423 sequences), MCG-15 (15.41%, 11,218 sequences), MCG-6 (10.70%, 7,428 sequences), MCG-5a (7.71%, 5,810 sequences) and MCG-11 (6.61%, 4,770 sequences). For subgroup MCG-4, the amount of distinct OTU is much higher in sediment of cyanobacteria-dominated area, although total OTU number and its sequence abundance is approximate at the two study areas (**Supplementary Table S2**). Besides, MCG-11 was more abundant in the sediment of cyanobacteria-dominated area, while MCG-6 and MCG-15 were significantly accumulated in sediment of lake zones dominated by macrophyte.

**FIGURE 5:**
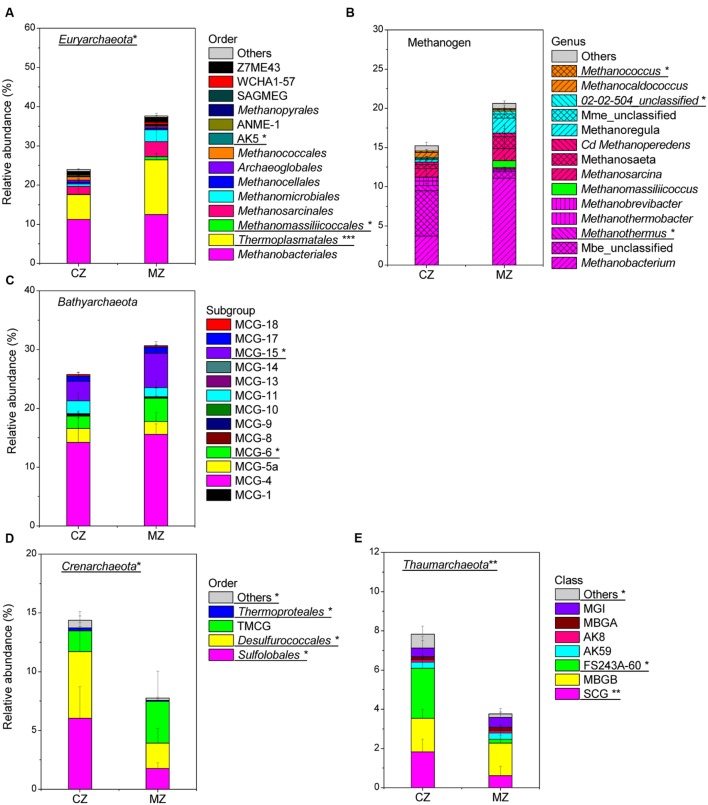
**Comparison of specific phylum composition of sediment Archaea between cyanobacteria-dominated zone (CZ) and macrophyte-dominated zone (MZ): **(A)** phylum *Euryarchaeota* (SAGMEG, South African Gold Mine Euryarchaeotal Group); **(B)** methanogen at genus level (magenta shadow stands for order *Methanobacteriales*, green stands for order *Methanomassiliicoccales*, pink stands for order *Methanosarcinales*, cyan stands for order *Methanomicrobiales*, orange indicated order *Methanococcales*; Cd, Candidatus; Mme, *Methanomicrobiaceae*; Mbe, *Methanobacteriaceae*) **(C)** phylum *Bathyarchaeota*; **(D)** phylum *Crenarchaeota* (TMCG, Terrestrial Miscellaneous Crenarchaeotal Group); **(E)** phylum *Thaumarchaeota* (no order information is available, SCG, Soil Crenarchaeotic Group; MBGB, Marine Benthic Group B; MBGA, Marine Benthic Group A; MGI, Marine Group I).** Archaeal lineages significantly different between the two regions were underlined with signs for significance: ^∗^ for 0.05 level, ^∗∗^ for 0.01 level and ^∗∗∗^for 0.001 level.

Further community composition analysis of *Euryarchaeota*, *Bathyarchaeota*, *Crenarchaeota*, *Thaumarchaeota* and methanogens allowed the identification of the key groups that contributed to the significant differences between the zone sediments (**Figure 5**; **Supplementary Table S1**). In the sediment of the cyanobacteria-dominated zones, the following archaeal lineages were significantly more abundant (percent of total archaeal sequences): methanogenic genera [*Methanothermus* (*p* = 0.014) and *Methanocaldococcus* (*p* = 0.019)] (**Figure 5B**), *Sulfolobales* (*p* = 0.018) and *Desulfurococcales* (*p* = 0.019) (**Figure 5D**), the Soil Crenarchaeotic Group (SCG, *p* = 0.006) and FS243A60 (*p* = 0.011) (**Figure 5E**). In the macrophyte-dominated zone sediment, *Thermoplasmatales* (*p* < 0.001) (**Figure 5A**), methanogenic genera *Methanomassiliicoccus* (*p* = 0.011) (**Figure 5B**), MCG-6 (*p* = 0.037) and MCG-15 (*p* = 0.045) (**Figure 5C**) were significantly more abundant. The relative abundance of the archaeal lineages (AK5, MCG-2, *Thermoproteales*, other *Crenarchaeota* and other *Thaumarchaeota*) was less than 0.3% of the total archaeal sequences, and they were not considered for further analysis, although their relative abundance was significantly different between the two zones’ sediments.

## Discussion

### Dominance of Archaeal Lineages Related to Sulfur Metabolism in Sediment of Lake Area with Planktonic Cyanobacteria

Previous studies have indicated that the degradation of settled biomass of cyanobacterial blooms at the sediment-water interface directly contributes to the availability of P, Fe, and S and induces bio-stimulated cycling of such elements (Handley et al., 2012; Zhu et al., 2013; Han et al., 2015). Interactions among sedimentary cycles of P, S, and Fe can influence their availability and mobility because their iron-containing forms tightly correlate with each other (Rozan et al., 2002). Moreover, severe cyanobacterial blooms can further induce local hypoxia (‘black blooms’), in which the dark water color probably results from the formation of transition metal sulfides such as FeS (Wang et al., 2014). Bacterial lineages *Desulfovibrio*, *Thiobacillus*, and *Sulfuricurvum*, which taking active part in sulfur metabolism are the dominant groups in the water column and the surface sediment of cyanobacteria-dominated zones (Li et al., 2012; Chen et al., 2015). Compared to the bacterial functional exploration, less information is available for the dominant archaeal lineages in this type of habitat. And the knowledge of archaeal groups participating in sulfur cycling is even more inadequate.

In this study, the archaeal lineages [*Crenarchaeota* (*Sulfolobales* and *Desulfurococcales*), *Aigarchaeota*, *Thaumarchaeota* (FS243A-60)] dominated in the sediment in the cyanobacteria-dominated zone (**Figures 3** and **4**). The analysis of single-cell genomes of several *Aigarchaeota* lineages indicated that these organisms might have the potential for anaerobic respiration using sulfite as the electron acceptor (Rinke et al., 2013). Members of *Sulfolobales* can use elemental sulfur to produce SO_4_^2-^ aerobically or H_2_S anaerobically (Suzuki et al., 2002; Plumb et al., 2007). In addition to those direct functional groups involved in sulfur cycling, the relative abundance of methanogenic genera such as *Methanothermus* and *Methanocaldococcus* was significantly higher in the cyanobacteria-dominated zone sediment (**Figure 5B**). Note that some species of *Methanothermus* and *Methanocaldococcus* could also participate in the sulfur cycle by using elemental sulfur, sulfite or thiosulfate as alternative sulfur sources (Liu et al., 2012; Susanti and Mukhopadhyay, 2012). Certainly, the specific role of those sulfur-related archaeal lineages is still an open question, but the current results strongly indicate that elementary cycling (P, Fe, and S) in the sediment of cyanobacterial bloom areas does not only depend on the bacterial population.

### Accumulation of Archaeal Lineages Involved in Complex Organic Carbon Metabolism in the Sediment of Macrophyte-Dominated Area

Organic matter from macrophytes has a higher C/N ratio and is regarded as less suitable for microbial transformation. In the macrophyte-dominated zone, a substantial portion of dead plant tissue continuously settles onto the sediment as litter. Long term accumulation of recalcitrant organic matter results in higher TOC and TN content in macrophyte-dominated zone sediment. Not only that, the concentrations of Chl*a*, LOI and TC/TN tended to be higher in the macrophyte-dominated zone sediment.

In this study, archaeal lineages [*Euryarchaeota* (*Thermoplasmatales*), *Woesearchaeota*] were significantly abundant in the macrophyte-dominated zone sediment, which might be a response of the archaeal community to the available substrate type (**Figures 4** and **5A**). The *Thermoplasmatales* in this study mainly match Marine Benthic Group D and Deep-sea Hydrothermal Vent Euryarchaeota Group-1 (MBG-D/DHVEG-1) (65.93% of total *Thermoplasmatales* sequences, significantly enriched in sediment of macrophyte dominated area, **Supplementary Figure S3**), which are usually detected in marine sediments around the world (Lloyd et al., 2013b). The analysis of single-cell genomes of MBG-D/DHVEG-1 indicated that this archaeal group encodes extracellular protein-degrading enzymes such as gingipain and clostripain (Lloyd et al., 2013b). Members of *Woesearchaeota*, which was previously named Deep-sea Hydrothermal Vent Euryarchaeota Group-6, might primarily be involved in anaerobic carbon cycling (Castelle et al., 2015). Additionally, *Woesearchaeota* has been detected as dominating in wastewater-treating bioreactors (Kuroda et al., 2015). The advantage of certain archaeal lineages in recalcitrant carbon utilization may provide them with supplementary niches which help to survive in sediment of macrophyte-dominated zones.

### Diversity of *Bathyarchaeota* in Lake Taihu Sediment and Their Segregation between Lake Sediment of Cyanobacteria- and Macrophyte-Dominated Area

Twenty eight percent of total archaeal sequences in this study belong to phylum *Bathyarchaeota*, which tended to be more abundant in the macrophyte-dominated zone sediment (**Figure 3**). Members of *Bathyarchaeota* are globally distributing in various marine and continental habitats, being recently detected in the anoxic organic-rich sediments of three freshwater karstic lakes (Fillol et al., 2015). Until now, no isolate of *Bathyarchaeota* has been cultivated or characterized. Previous studies have suggested that *Bathyarchaeota* are composed of anaerobic heterotrophs that do not participate in methane and sulfur cycles but likely use refractory organic carbon such as detrital proteins and aromatic compounds (Biddle et al., 2006; Lloyd et al., 2013b; Meng et al., 2014).

More than half of the defined MCG subgroups could be detected in Lake Taihu. Members of the highly diverse *Bathyarchaeota* were divided into 17 subgroups (Kubo et al., 2012), which were then extended to 21 subgroups (Fillol et al., 2016). In this study, MCG-4 was the dominant subgroup in both regions, while inner subgroup diversity were much higher in the sediment of cyanobacteria-dominated zones (**Supplementary Table S2**). Despite MCG-4 are mainly found in marine sediments, they might probably be also recovered from freshwater or brackish lakes (Gagen et al., 2013). Culture treatments of sediment core from White Oak River estuary revealed that members of MCG-8 and MCG-4 clusters are amenable to *in vitro* growth in synthetic medium (Gagen et al., 2013). Furthermore, unlike other strict anaerobes, members of these affiliations might not be killed by oxygen after a very short exposure time (e.g., <2 h in some cases, Gagen et al., 2013).

MCG-6 (*p* = 0.037) and MCG-15 (*p* = 0.045) were significantly abundant in the sediment of macrophyte-dominated zones. In three freshwater karstic lakes, subgroups MCG-5a and -5b appeared as planktonic specialists, MCG-6 emerged as a generalist group, and subgroup MCG-15 was prevalent in cDNA-based datasets of organic-rich sediments (Fillol et al., 2015). MCG-15, previously named Group C3, has been mostly detected in marine sediment (Inagaki et al., 2001, 2003, 2006), in which this group was found to be metabolically active uptaking acetate (Hugoni et al., 2015; Na et al., 2015). Undoubtedly, the spatial-temporal variation of such diverse MCG subgroups needs to be further investigated to uncover their roles in each featured zones.

## Conclusion

Our work revealed significant heterogeneity of the sediment Archaea of two primary producer (Cyanobacteria vs. macrophyte)-dominated zones of Lake Taihu. Besides the phylum level difference, sulfur metabolism-related archaeal lineages, such as *Sulfolobales* and *Desulfurococcales*, accumulated in the sediment of algae-dominated area. In the macrophyte-dominated zone sediment, complex organic carbon degradation-related archaeal lineages, such as *Thermoplasmatales*, might be the main contributors to the carbon cycle. Among the abundant *Bathyarchaeota*, 13 subgroups were identified with MCG-4 dominant in both regions, while MCG-6 and MCG-15 were significantly abundant in the sediment of macrophyte-dominated zones. Nevertheless, more work is needed to investigate the coordination of archaeal and bacterial functional groups in the crucial processes of matter and element cycling in this shallow lake. The present study could contribute to our understanding of the roles of archaeal communities in the biogeochemical cycles of lake sediment.

## Author Contributions

XF did the sampling job and data analysis. She wrote most parts of the manuscript. PX made the contribution in the experimental design, data demonstration and the draft revision.

## Conflict of Interest Statement

The authors declare that the research was conducted in the absence of any commercial or financial relationships that could be construed as a potential conflict of interest.

## Footnotes

1 http://huttenhower.sph.harvard.edu/galaxy
