# Supplementary material for: Differences in the Composition of Archaeal Communities in Sediments from Contrasting Zones of Lake Taihu
Source: Front Microbiol. 2016 Sep 21;7:1510. doi: 10.3389/fmicb.2016.01510 (PMC5030832; doi:10.3389/fmicb.2016.01510)
Supplement: Supplementary file 1 [file Data_Sheet_1.DOCX]

Supplementary Material

Differences in the Composition of Archaeal Communities in Sediments from Contrasting Zones of Lake Taihu

**Xianfang Fan^1,2†^, Peng Xing^1*†^**

^1^State Key Laboratory of Lake Science and Environment, Nanjing Institute of Geography & Limnology, Chinese Academy of Sciences, Nanjing 210008, China

^2^ State Key Laboratory of Soil and Sustainable Agriculture, Institute of Soil Science, Chinese Academy of Sciences, Nanjing 210008, China

*** Correspondence:** Dr. Peng Xing: [pxing@niglas.ac.cn](mailto:pxing@niglas.ac.cn)

**^†^** These authors have contributed equally to this work

**Table S1.** Results of normality test and difference test of multiple variables between cyanobacteria-dominated and macrophyte-dominated zones shown in Fig.3a, Fig. 5, Table 1 and Table 2 (data distribution was tested by Kolmogorov-Smirnov method; if it was normal, T test was applied for the significance test, otherwise, Mann-Whitney test was applied; significant level was set as 0.05).

| Variable | Test of  [normality](http://www.baidu.com/link?url=QrvgMezE1p5YT40PNBtLHugaLUQtpmBD9zRuzuXOrjuaThS_muT93khgbiMUEu6Y7YjhQUjvYzhFR1IfbCeaGlFqxVxFtQlqwX6MwVQjoXJpA8qsX1Ln8k3bbCWtLqOS) | Test of difference |  | Variable | Test of  [normality](http://www.baidu.com/link?url=QrvgMezE1p5YT40PNBtLHugaLUQtpmBD9zRuzuXOrjuaThS_muT93khgbiMUEu6Y7YjhQUjvYzhFR1IfbCeaGlFqxVxFtQlqwX6MwVQjoXJpA8qsX1Ln8k3bbCWtLqOS) | Test of difference |
| --- | --- | --- | --- | --- | --- | --- |
| **Data in Figure 3a**  Aenigmarchaeota | 0.000 | 0.584 |  | MCG-17 | 0.200 | 0.512 |
| Aigarchaeota | 0.200 | 0.003 |  | MCG-18 | 0.046 | 0.715 |
| Archaea_Unclassified | 0.180 | 0.745 |  | **Data in Figure 5c** |  |  |
| Crenarchaeota | 0.200 | 0.027 |  | Sulfolobales | 0.014 | 0.018 |
| Diapherotrites | 0.005 | 0.100 |  | Desulfurococcales | 0.200 | 0.019 |
| Euryarchaeota | 0.200 | 0.017 |  | TMCG | 0.200 | 0.193 |
| Korarchaeota | 0.001 | 0.715 |  | Thermoproteales | 0.200 | 0.024 |
| MHVG-1 | 0.200 | 0.005 |  | Other Cren | 0.004 | 0.028 |
| MHVG | 0.016 | 0.269 |  | **Data in Figure 5d**  SCG | 0.200 | 0.006 |
| Bathyarchaeota | 0.200 | 0.350 |  | MBGB | 0.200 | 0.813 |
| MEG | 0.002 | 0.018 |  | FS243A-60 | 0.038 | 0.011 |
| Parvarchaeota | 0.048 | 0.273 |  | AK59 | 0.200 | 0.974 |
| SM1K20 | 0.130 | 0.161 |  | AK8 | 0.061 | 0.789 |
| TVG8AR30 | 0.088 | 0.318 |  | MBGA | 0.200 | 0.503 |
| Thaumarchaeota | 0.200 | 0.009 |  | MGI | 0.027 | 0.715 |
| Woesearchaeota  **Data in Figure 5a** | 0.133 | 0.048 |  | Other Thaum | 0.140 | 0.027 |
| Thermoplasmata | 0.502 | <0.001 |  | **Data in Figure 5e**  methanogen | 0.200 | 0.338 |
| Methanobacteriales | 0.200 | 0.775 |  | Methanobacterium | 0.088 | 0.055 |
| Thermoplasmatales | 0.070 | <0.001 |  | Mbe_unclassified | 0.003 | 0.068 |
| Methanosarcinales | 0.123 | 0.232 |  | Methanothermus | 0.200 | 0.014 |
| Methanomicrobiales | 0.015 | 0.273 |  | Mthermobacter | 0.018 | 0.100 |
| Methanocellales | 0.007 | 0.584 |  | Mbrevibacter | 0.011 | 0.100 |
| Archaeoglobales | 0.200 | 0.711 |  | Methanosarcina | 0.006 | 0.361 |
| Methanococcales | 0.170 | 0.069 |  | Methanoregula | 0.002 | 0.273 |
| AK5 | 0.001 | 0.045 |  | Methanosaeta | 0.048 | 0.201 |
| ANME-1 | 0.020 | 0.201 |  | Cd Mperedens | 0.055 | 0.530 |
| Methanopyrales | 0.031 | 0.100 |  | Mme_unclassified | 0.002 | 0.361 |
| SAGMEG | 0.200 | 0.466 |  | 02-02-504_un | 0.200 | 0.146 |
| WCHA1-57 | 0.021 | 0.200 |  | Mcaldococcus | 0.200 | 0.019 |
| Z7ME43 | 0.102 | 0.137 |  | Methanococcus | 0.200 | 0.368 |
| Other Eury | 0.098 | 0.124 |  | Mmassiliicoccus | 0.001 | 0.011 |
| **Data in Figure 5b**  MCG-1 | 0.039 | 0.170 |  | Others | 0.195 | 0.965 |
| MCG-4 | 0.200 | 0.564 |  | **Data in Table 1**  Chla | 0.200 | 0.407 |
| MCG-5a | 0.200 | 0.860 |  | TOC | 0.200 | 0.003 |
| MCG-6 | 0.108 | 0.037 |  | TN | 0.200 | 0.003 |
| MCG-8 | 0.004 | 0.465 |  | TP | 0.087 | 0.504 |
| MCG-9 | 0.122 | 0.060 |  | LOI | 0.001 | 0.465 |
| MCG-10 | 0.200 | 0.796 |  | TC/TN | 0.523 | 0.210 |
| MCG-11 | 0.200 | 0.461 |  | **Data in Table 2**  OTUs | 0.189 | 0.468 |
| MCG-13 | <0.001 | 0.361 |  | Chao | 0.744 | 0.760 |
| MCG-14 | <0.001 | 0.361 |  | Shannon | 0.381 | 0.076 |
| MCG-15 | 0.200 | 0.045 |  | PD | 0.711 | 0.354 |

MHVG-1: Marine Hydrothermal Vent Group 1; MHVG: Marine Hydrothermal Vent Group; MEG: Miscellaneous Euryarchaeotic Group; SAGMEG: South African Gold Mine Euryarchaeotal Group; Other Eury: Other Euryarchaeota; TMCG: Terrestrial Miscellaneous Crenarchaeotal Group; Other Cren: Other Crenarchaeota; SCG: Soil Crenarchaeotic Group; MBGB: Marine Benthic Group B; MBGA: Marine Benthic Group A; MGI: Marine Group I ; Other Thaum: Other Thaumarchaeota; Mbe: *Methanobacteriaceae*; Mthermobacter: Methanothermobacter; Mbrevibacter: Methanobrevibacter; Cd Mperedens: Candidatus Methanoperedens; Mme: *Methanomicrobiaceae*; 02-02-504_un: 02-02-504_unclassified ; Mcaldococcus: Methanocaldococcus; Mmassiliicoccus: Methanomassiliicoccus.

**Table S2**. The total OTU & distinct OTU number of each bathyarchaeal subgroup in a given region and their average relative abundance (RA, percentage of sequences from a certain subgroup in total *Bathyarchaea*; standard deviation of replicates are in brackets).

| Subgroups | Cyanobacteria-dominated zone | | |  | Macrophyte-dominated zone | | |
| --- | --- | --- | --- | --- | --- | --- | --- |
|  | OTUs | distinct OTUs | Average  RA (%) |  | OTUs | distinct OTUs | Average  RA (%) |
| MCG-1 | 1 | 0 | 0.09 (0.13) |  | 2 | 1 | 0.02 (0.02) |
| MCG-4 | 389 | 84 | 56.42 (4.82) |  | 336 | 31 | 51.05 (5.07) |
| MCG-5a | 19 | 3 | 8.48 (3.79) |  | 18 | 2 | 6.78 (4.11) |
| MCG-6 | 9 | 0 | 8.36 (3.53) |  | 9 | 0 | 13.51 (7.09) |
| MCG-8 | 19 | 5 | 1.00 (1.48) |  | 18 | 4 | 0.34 (0.38) |
| MCG-9 | 1 | 0 | 0.59 (0.41) |  | 1 | 0 | 0.12 (0.20) |
| MCG-10 | 8 | 2 | 0.35 (0.25) |  | 6 | 0 | 0.27 (0.28) |
| MCG-11 | 26 | 4 | 8.16 (3.89) |  | 22 | 0 | 4.75 (3.29) |
| MCG-13 | 1 | 1 | 0.01 (0.01) |  | 0 | 0 | 0.00 (0.00) |
| MCG-14 | 1 | 1 | 0.01 (0.02) |  | 0 | 0 | 0.00 (0.00) |
| MCG-15 | 47 | 2 | 12.53 (4.19) |  | 56 | 11 | 18.86 (5.30) |
| MCG-17 | 31 | 7 | 2.99 (1.52) |  | 27 | 3 | 3.56 (0.83) |
| MCG-18 | 1 | 0 | 1.01 (0.55) |  | 1 | 0 | 0.74 (0.57) |
| **Total** | **553** | **109** | **45.64 (18.32)** |  | **496** | **52** | **54.36 (7.71)** |

**Figure S1.**Variation of overlying water physicochemical properties at the eleven sampling sites, among which S1 to S6 were located in cyanobacteria-dominated zone (CZ) and S7 to S11in macrophyte-dominated zone (MZ).

**Figure S2.** Abundance of Archaea and Bacteria evaluated by qPCR method: (a) archaeal and bacterial 16S rRNA gene copies at the eleven sampling sites; (b) average archaeal 16S rRNA gene copies at CZ and MZ (c) average bacterial 16S rRNA copies at CZ and MZ (CZ: cyanobacteria-dominated zone; MZ: macrophyte-dominated zone).

**Figure S3.** Results of Linear Discriminant Analysis(LDA) using LefSe software with LDA values of 4.0 or higher: a. Bar plot indicating microbial groups within the two zone sediments; b. Cladogram plot indicating the phylogenetic distribution of microbial lineages associated with the two zone sediments (CZ: cyanobacteria-dominated zone; MZ: macrophyte-dominated zone; MBG_D: Marine Benthic Group D/Deep Sea Hydrothermal Vent Group-1; GC: Group C3; FS: FS243A-60; AIS: Aigarchaeota Incertae Sedis; un: unclassified).
